# A bibliometric analysis in the fields of preventive medicine, occupational and environmental medicine, epidemiology, and public health

**DOI:** 10.1186/1471-2458-6-301

**Published:** 2006-12-15

**Authors:** Elpidoforos S Soteriades, Matthew E Falagas

**Affiliations:** 1 Alfa Institute of Biomedical Sciences (AIBS), Athens, Greece; 2 Department of Environmental Health, Occupational Health Program, Harvard School of Public Health, Boston, Massachusetts, USA; 3 Department of Medicine, Tufts University School of Medicine, Boston, Massachusetts, USA

## Abstract

**Background:**

Research in the fields of Preventive Medicine, Occupational/Environmental Medicine, Epidemiology and Public Health play an important role in the advancement of knowledge. In order to map the research production around the world we performed a bibliometric analysis in the above fields.

**Methods:**

All articles published by different world regions in the above mentioned scientific fields and cited in the Journal Citation Reports (JCR) database of the Institute for Scientific Information (ISI) during the period 1995 and 2003, were evaluated. The research production of different world regions was adjusted for: a) the gross domestic product in 1995 US dollars, and b) the population size of each region.

**Results:**

A total of 48,861 articles were retrieved and categorized. The USA led the research production in all three subcategories. The percentage of articles published by USA researchers was 43%, 44% and 61% in the Preventive Medicine, Epidemiology, and Public Health subcategories, respectively. Canada and Western Europe shared the second position in the first two subcategories, while Oceania researchers ranked second in the field of Public Health.

**Conclusion:**

USA researchers maintain a leadership position in the production of scientific articles in the fields of Preventive Medicine, Occupational/Environmental Medicine and Epidemiology, at a level similar to other scientific disciplines, while USA contribution to science in the field of Public Health is by all means outstanding. Less developed regions would need to support their researchers in the above fields in order to improve scientific production and advancement of knowledge in their countries.

## Background

The fields of Preventive Medicine, Occupational/Environmental Medicine, Epidemiology and Public Health constitute scientific fields, along with clinical medicine, which play an important role on people's health around the world [1-3]. Research performed in the above fields provides the basis for identifying significant health problems in the population and supports the development of knowledge-based interventions to educate people on health issues, promote health, and protect people's vulnerability to different health hazards [4-6]. Furthermore, published scientific articles on such topics promote public dialogue and provide opportunities for policy makers to address public health issues through legal interventions [7,8].

While, the academic community strives to identify the best approach to assess the quantity and quality of research production between different geographical boundaries, languages and scientific disciplines, most scientists agree on the utility of the impact factor as well as additional adjusted indicators for international comparisons [9]. Several bibliometric analyses have been published in the medical literature on different topics [10-13]. Our group has also previously published a number of articles assessing the research production of different world regions on several scientific disciplines, however there are limited data on the bibliometric assessment of research in the fields of our present study with several reported limitations [14-22].

For example, Gehanno JF et al has reported that 1.4% of journals in the field of Occupational Health (a total of 8 journals) account for 27% of published articles in the field, while Navarro A et al have reported that one or more institutions in the United States contributed over 40% of articles in Occupational Health [18,19]. In addition, Verbeek J et al have studied the sensitivity and specificity of search terms in identifying Occupational Health intervention studies [20].

In the current investigation, we sought to identify and quantify the research production of different world regions in the fields of Preventive Medicine, Occupational and Environmental Medicine, Epidemiology, and Public Health around the world.

## Methods

The methodology we used in our study parallels other bibliometric studies performed by our research group in order to evaluate research productivity in specific scientific disciplines [14-17].

### Data sources

We searched for articles published between 1995 and 2003 and included in the category of the "Preventive and Occupational Medicine, Epidemiology and Public Health", of the Journal Citation Reports (JCR) database of the Institute for Scientific Information (ISI) and the PubMed database [23,24]. At the time of our analysis, ISI provided the electronic list of the scientific journals for each category examined, only for the years 1999 – 2003, while for the period 1995 – 1998, the ISI database provided only the impact factor of each scientific journal. Therefore, for the years 1995 – 1998 we identified the same journals that were included in each corresponding category in the year 1999 and we reviewed them and included them in the analysis as long as the impact factor for each year examined was available.

### Journals and scientific fields

The articles' origin was assigned by searching the address of the first author of each article, which is registered in the PubMed database. For each search in the PubMed database, a phrase consisting of four parts joined together by the so-called Boolean operators, i.e. AND, OR, and NOT was used in the search field. Moreover, each separate search was limited to a specific year. Publication types such as letters, editorials, and news reports were excluded from the analysis. The results of these searches (the number of articles produced by each world region in a specific journal within a year) were summed up.

Out of 89 journals included in the journal category investigated during the period 1999–2003, a total of 72 journals were included in our study. A further division of these journals into three subcategories was performed; the first subcategory included journals with main focus on Preventive, Occupational and Environmental Medicine (28 journals), the second on Epidemiology (16 journals), and the third on Public Health (28 journals).

### World regions

For the purpose of our study, the world was divided into 9 regions; United States of America (USA), Canada, Western Europe, Japan, Oceania, Asia (excluding Japan), Eastern Europe, Latin America and the Caribbean, and Africa; a classification based on a combination of geographic, economic and scientific criteria.

### Article retrieval

The sum of articles produced by all different world regions in a journal was compared to the actual total number of articles published in that journal for a specific year. This number was obtained again from PubMed by not using any address limits. In this way we were able to check for missed or unretrieved addresses. We considered a search result acceptable if less than 5% of the total articles of a specific journal during a year were missed by our methodology, otherwise we performed manual searches for the author's address. To strengthen the methodological validity of our study, two investigators independently performed data collection. In cases of disagreement between the two investigators the results were discussed in meetings of all investigators of our research group.

### Data analyses

The index of quantity of research productivity was the number of published articles. The quality of research productivity was estimated by the mean impact factor of the examined journals for each published article. Finally, the product of the number of articles published in a journal multiplied by the corresponding impact factor of its journal, for each year studied, was considered as an integrated index of the quantity and quality of research productivity. The sum of these products from all journals, for each world region within a year, was assigned as a "total research product" for each world region.

In order to evaluate factors possibly associated with the research published in Preventive and Occupational/Environmental Medicine, Epidemiology, and Public Health journals, we used relevant "World Development Indicators" from the online databases of the World Bank [25]. The research productivity of different world regions (estimated by the "total product") was adjusted for: a) the gross domestic product in standard 1995 US dollars, and b) the population size of each region.

## Results

Using the methodology described above, we managed to retrieve and categorize 48,681 out of 49,980 articles, (97.4%) from the implicated journals indexed in PubMed during the study period. In tables 1, 2, and 3 we present the actual number of articles produced each year during the study period from each world region, in the "Preventive and Occupational/Environmental Medicine", "Epidemiology", and "Public Health" ISI categories, respectively. The last two columns of each table present data adjusted for the gross domestic product (GDP) and the population size of each region.

As shown in Table 1, the USA is the world leader in research productivity in the "Preventive and Occupational/Environmental Medicine" subcategory, in terms of both quantity and quality (7,280 articles, mean impact factor 1.5) of published papers. Researchers in the USA published more than one third of articles in this field. Western Europe comes second in the number of published articles (5,306), while Canada ranks second regarding the mean impact factor (1.4) along with Central and Latin America. When adjusted for GDP, Canada is the most productive region, whereas USA reclaims it leadership when data are adjusted for population size.

Similar rankings for USA, Canada, and Western Europe are seen in Table 2, referring to articles published in the field of epidemiology. It is notable that researchers in Europe publish more articles in this field compared to their own research productivity in the Preventive Medicine and Occupational Medicine subcategory, while Canada researchers rank again second when research production is adjusted for gross domestic product.

Finally, data from the 28 Public Health journals are presented in Table 3. USA again leads the research production, and there is a much bigger gap compared to the second region (Western Europe). USA researchers rank first regarding both raw and adjusted data and produce about two thirds (61%) of all published articles around the world. We also note that researchers from Oceania rank first in this subcategory, together with the USA, with respect to the mean impact factor, and come second when total product is adjusted for both gross domestic product and population size.

In all of the 3 subcategories examined, the annual number of published articles worldwide showed an increasing trend, a phenomenon that was more pronounced in the "Public Health" subcategory (Table 3), probably due to the gradual increase in the number of journals of the ISI category throughout the study period. After accumulating the data from all 3 subcategories (data not shown), USA came first in all indices, namely the number of articles (23,918), the mean impact factor (1.96), the total product (46,879), and the total product per GDP (61.1), and per million of population (167).

## Discussion

In our study we have quantified the research production around the world in several related public health disciplines as well as in Occupational and Environmental Medicine looking at a specific time period and adjusting our findings from different world regions for their corresponding gross domestic product and population size.

We found that researchers from the USA maintain a leadership position in the publication of scientific articles in the fields of Preventive Medicine, Occupational/Environmental Medicine, Epidemiology and Public Health similar to other scientific fields examined. The most noteworthy finding of our study is the fact that USA researchers contributed more than 60% of all articles published in the field of Public Health around the world (indexed in the reviewed databases). Even after overall adjustment for gross domestic product and population size, USA continued to lead. However, Canada researchers ranked first when scientific production was adjusted for gross domestic product in the fields of Preventive Medicine, Occupational/Environmental Medicine and Epidemiology. Furthermore, we found that four regions of the world with more than 50% of the world's population have minimal contribution in the scientific fields studied.

Our results support previous findings suggesting that the majority of research published in scientific journals is carried out in the developed world [18]. It is also notable that USA evolved as the strongest leader in the field, since it demonstrated the highest productivity in most fields examined. The above finding is not a surprise given the long tradition of agencies and institutions in the USA in implementing research and population-based public health programs [26,27]. Similar observations with respect to public health research would also apply to Canada [28]. In addition, our study reveals a significant gap in the participation of certain world regions in scientific research in the above fields examined at least with respect to the published literature in the English language [29-31].

Several limitations of our current investigation have been cited previously and would also apply [14-17]. Most of the limitations are related to the databases used to retrieve articles as they consist largely of English-language journals therefore possibly contributing to selection bias due to language barriers. In addition, the above databases do not represent all scientific and biomedical journals published. Other limitations include the incorrect citation of origin for the authors, and the use of impact factors as an indicator of research quality [32]. Finally, many articles of public health importance appear in journals other than those we included in our searched categories. Nevertheless, we believe that comparisons made even under known or unknown limitations, are useful and could provide important insight with respect to the direction, amount, and impact of research productivity around the world [33].

## Conclusion

In summary, we found that the USA, Western Europe, Canada and Oceania are the leading regions in the research production in the fields of Preventive/Occupational/Environmental Medicine, Epidemiology and Public Health, with the USA being far ahead especially in the subcategory of Public Health. We believe that our findings clearly indicate the need to promote research in the above fields in less developed regions of the world. Promoting research in less developed areas such as Africa, Asia, Latin America and Eastern Europe, may involve but not limited to the development of infrastructure including research and academic institutes, the improvement of current collaborative partnerships with developed nations, increased sponsorship and support from world agencies such as the World Health Organization and the United Nations, and the implementation of programs including free access to online journals and translation services for scientific articles published in languages other than the English language. All of the above are likely to increase research and development in the less developed regions of the world and improve peoples' health through the diffusion of knowledge and the implementation of large-scale research projects.

## Abbreviations

**ISI **– Institute for Scientific Information

**GDP **– Gross Domestic Product

**JCR **– Journal Citation Reports

## Competing interests

The author(s) declare that they have no competing interests.

## Authors' contributions

MEF had the idea for the study and developed the methodology in collaboration with ESS. Both authors supervised the data collection, analysis and interpretation. Also, both authors contributed to the writing of the manuscript and approved its final version.

The authors would like to thank Ms. Paraskevi A. Papastamataki, RN, and Dr. Ioannis A. Bliziotis, MD, for their help in the collection and analysis of data.

## Pre-publication history

The pre-publication history for this paper can be accessed here:



## Figures and Tables

**Table 1:** Data regarding the worldwide research production in the "Preventive and Occupational/Environmental Medicine" subcategory.

	**1995**	**1996**	**1997**	**1998**	**1999**	**2000**	**2001**	**2002**	**2003**	**Total number of articles for the whole period**	**Percentage of worldwide production (articles)**	**Mean impact factor of all articles**	Total product for the whole period *	**Total product/gross domestic product †**	**Total product/millions of population**
**USA**	777	826	883	804	834	885	799	752	720	7,280	43.4	1.5	10,623	13.8	37.8
**Canada**	97	95	94	88	86	84	90	72	81	787	4.7	1.4	1,094	18.2	36.0
**Western Europe**	500	502	608	571	659	574	582	555	755	5,306	31.6	1.3	6,775	7.5	17.4
**Japan**	71	110	118	130	106	125	108	112	135	1,015	6.1	1.0	1,011	2.0	8.0
**Oceania**	15	18	23	23	37	38	53	41	39	287	1.7	1.2	331	7.1	11.0
**Asia**	91	103	121	92	141	133	161	165	259	1,266	7.6	1.0	1,265	3.9	0.4
**Eastern Europe**	20	26	39	54	38	46	51	77	110	461	2.8	1.1	525	5.3	1.2
**Central and Latin America**	20	17	30	20	14	39	30	34	48	252	1.5	1.4	345	2.1	0.7
**Africa**	11	9	13	15	11	12	16	23	18	128	0.8	1.1	137	2.8	0.2
**TOTAL (or average)**	1,602	1,706	1,929	1,797	1,926	1,936	1,890	1,831	2,165	16,782	-	-	-	(7.6)	-

* Defined as the product of articles multiplied by the corresponding impact factor of their journal

**† **Total product (defined by the number of articles multiplied by the impact factor of the journal) divided by gross domestic product in tens of trillions of 1995 US dollars

**Table 2:** Data regarding the worldwide research production in the "Epidemiology" subcategory.

	**1995**	**1996**	**1997**	**1998**	**1999**	**2000**	**2001**	**2002**	**2003**	**Total number of articles for the whole period**	**Percentage of worldwide production (articles)**	**Mean impact factor of all articles**	Total product for the whole period *	**Total product/gross domestic product †**	**Total product/millions of population**
**USA**	663	691	724	681	821	896	976	815	839	7,106	44.0	2.8	20,029	26.1	71.3
**Canada**	64	70	73	66	107	93	92	76	91	732	4.5	2.4	1776	29.5	58.4
**Western Europe**	621	634	664	686	694	754	814	649	784	6,300	39.0	2.0	12,347	13.7	31.8
**Japan**	21	19	26	22	35	36	48	32	36	275	1.7	2.3	643	1.3	5.1
**Oceania**	39	51	45	46	56	51	52	68	69	477	3.0	2.2	1,049	22.4	34.6
**Asia**	43	67	65	69	57	84	73	75	104	637	3.9	2.1	1,321	4.1	0.4
**Eastern Europe**	19	20	20	24	17	31	26	11	40	208	1.3	1.3	266	2.7	0.6
**Central and Latin America**	25	21	17	18	31	31	50	36	42	271	1.7	2.1	558	3.3	1.1
**Africa**	16	17	14	16	27	15	22	18	20	165	1.0	1.7	280	5.7	0.4
**TOTAL (or average)**	1,511	1,590	1,648	1,628	1,845	1,991	2,153	1,780	2,025	16,171	-	-	-	(13.1)	-

* Defined as the product of articles multiplied by the corresponding impact factor of their journal

**† **Total product (defined by the number of articles multiplied by the impact factor of the journal) divided by gross domestic product in tens of trillions of 1995 US dollars

**Table 3:** Data regarding the worldwide research production in the "Public Health" subcategory.

	**1995**	**1996**	**1997**	**1998**	**1999**	**2000**	**2001**	**2002**	**2003**	**Total number of articles for the whole period**	**Percentage of worldwide production (articles)**	**Mean impact factor of all articles**	Total product for the whole period *	**Total product/gross domestic product †**	**Total product/millions of population**
**USA**	840	919	852	1,021	950	1,196	1,252	1,195	1,307	9,532	60.6	1.7	16,254	21.2	57.9
**Canada**	33	43	50	44	58	45	46	47	71	437	2.8	1.4	629	10.4	20.7
**Western Europe**	353	367	367	400	395	518	611	505	618	4,134	26.3	1.1	4,651	5.2	12.0
**Japan**	6	7	11	11	19	9	12	14	14	103	0.7	1.2	126	0.2	1.0
**Oceania**	28	28	30	37	42	57	36	61	111	430	2.7	1.7	711	15.2	23.5
**Asia**	31	40	34	52	62	63	85	91	91	549	3.5	1.4	753	2.3	0.2
**Eastern Europe**	3	5	3	10	6	15	15	21	24	102	0.7	1.2	125	1.3	0.3
**Central and Latin America**	11	16	10	15	19	18	23	17	34	163	1.0	1.6	265	1.6	0.5
**Africa**	17	21	22	22	27	24	52	38	55	278	1.8	1.6	457	9.3	0.6
**TOTAL (or average)**	1,322	1,446	1,379	1,612	1,578	1,945	2,132	1,989	2,325	15,728	-	-	-	(8.2)	-

* Defined as the product of articles multiplied by the corresponding impact factor of their journal

**† **Total product (defined by the number of articles multiplied by the impact factor of the journal) divided by gross domestic product in tens of trillions of 1995 US dollars
